# Case from Ballarat Base Hospital, Victoria - Epididymal cavernous haemangioma mimicking testicular torsion

**DOI:** 10.1016/j.eucr.2025.103261

**Published:** 2025-11-01

**Authors:** Siyu Huang, Thomas McLean, Annjaleen Hansa, Jordan Santucci, Andrew Silagy, Lydia Johns-Putra

**Affiliations:** a Grampians Health Ballarat, VIC, Australia; b Dorevitch Pathology Ballarat, VIC, Australia

**Keywords:** Epididymal AND cavernous AND haemangioma, Vascular AND malformation, Paratesticular AND mass

## Abstract

Epididymal haemangioma represents a rare aetiology of paratesticular masses. Here, we report a case of a 15-year-old adolescent who presented with acute onset left testicular pain and swelling. The patient underwent urgent scrotal exploration, which revealed a necrotic left testis, leading to subsequent orchidectomy. Pathological examination revealed a diffusely haemorrhagic testis with irregular collection of dilated vessels throughout the epididymis, in keeping with a cavernous haemangioma. This case highlights epididymal cavernous haemangioma as a rare cause of acute scrotal symptoms mimicking torsion.

## Highlights

• Cavernous haemangiomas are vascular malformations where leakage is prone to occur.
• There are very few reported cases of epididymal cavernous haemangioma.
• The onset of symptoms can be acute or insidious, mimicking various testicular conditions.
• Secondary intratesticular haemorrhage can significantly impair the viability of the testis.

## 1 Background

Epididymal haemangioma is a rare, benign cause of paratesticular masses. The differential diagnosis of these masses includes malignant causes such as rhabdomyosarcoma, liposarcoma and leiomyosarcoma, as well as benign conditions such as lipoma, leiomyoma and haemangioma.^1^ Cavernous haemangiomas are vascular malformations composed of dilated blood vessels that resemble ‘caverns’ in which blood flow is impaired and leakage is prone to occur.^2^ There are only five reported cases of epididymal cavernous haemangioma, two of which involved both epididymal and testicular haemangioma. Orchidectomy was performed in most cases with the exception of one case in which epididymectomy was feasible. We present a case of paediatric epididymal cavernous haemangioma, illustrating our management approach and clinical reasoning.

## 2 Case presentation

We report a case of a 15-year-old adolescent who presented with acute onset left testicular pain and swelling. The patient developed sudden onset left testicular pain that woke him up from sleep. He presented to our Emergency Department more than 24 hours later due to worsening pain, redness, and swelling over the left testicle. The patient had constant pain in the left testicle with no radiation of the pain. He had no nausea or vomiting. The patient had no significant medical history. There was no trauma to the testicle. Physical examination revealed an erythematous, firmly swollen left testis with global tenderness but normal lie. Urine dipstick was negative for leukocytes, nitrites and blood. The patient underwent urgent scrotal exploration due to concern for testicular torsion. Ultrasound was not performed preoperatively due to the high clinical suspicion of testicular torsion.

Intraoperatively, the left testis was found to be in a normal anatomical position with an intact gubernaculum, not twisted, but it was necrotic, haemorrhagic, and very firm. A decision was made to perform left orchidectomy as the testis was not viable. Testicular tumour markers were sent as there was a concern for malignancy.

**Fig. 1:**
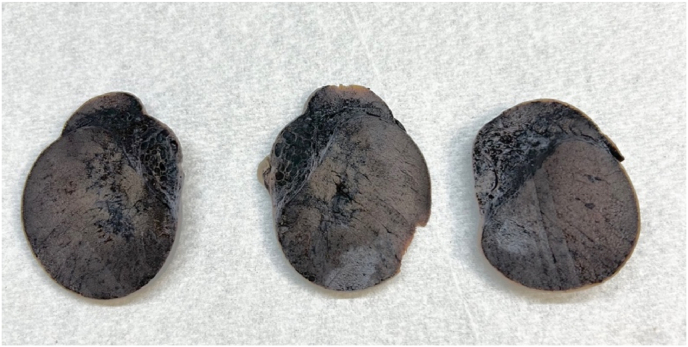
Serial sagittal sections of the formalin-fixed left testis demonstrating a homogenous brown cut surface and multiple cavitating cysts within the epididymis.

**Fig. 2:**
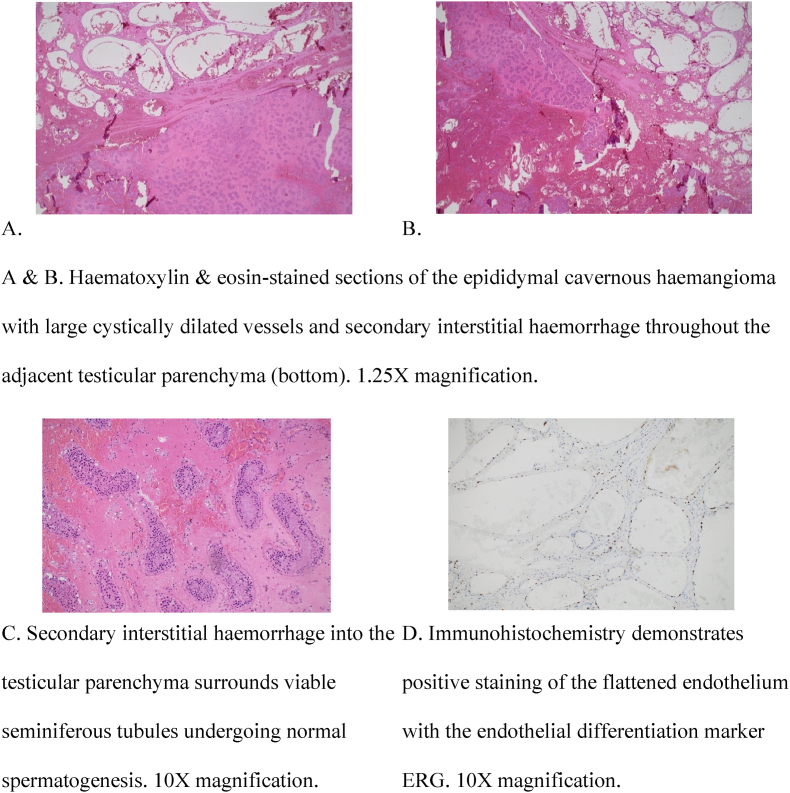
Microscopic features of left testicular specimen.

Dissection of the orchidectomy specimen revealed a firm, homogenous and diffusely haemorrhagic testis measuring 45 x 42 × 41mm and weighing 30g (Fig. 1). Histological examination demonstrated an irregular collection of cystically dilated vessels throughout the epididymis joined by anastomosing fibrous septations. The vessels were lined by a flattened endothelium with no atypia in keeping with a cavernous haemangioma. Degenerative epididymal tubules with epithelial necrosis were noted adjacent to the vascular lesions. However, in sections away from the vascular lesion, the majority of seminiferous tubules showed normal spermatogenesis with interstitial haemorrhage (Fig. 2). There was no evidence of malignancy. The patient's three-week postoperative follow-up showed an uneventful recovery. Tumour markers were negative, and an ultrasound demonstrated a healthy right testicle and epididymis.

## 3 Discussion and conclusions

Our report illustrates a case of epididymal cavernous haemangioma in a teenager who presented with acute onset testicular pain and swelling. Prompt scrotal exploration was performed, as the presentation was concerning for testicular torsion given the acute symptoms, examination findings, and patient demographics. In 1954, Robertson et al. described a similar presentation in a 15-year-old boy who presented five days after being awakened by scrotal pain and the development of a testicular mass.^3^ Immediate scrotal exploration was also performed due to concern for torsion.^3^ In their case, almost the entire testicle and epididymis were replaced by endothelial-lined spaces, and a diagnosis of testicular and epididymal haemangioma was made.^3^ In other reported cases, the presentations were more insidious.^4^^,^^5^ In two cases, an ultrasound was performed prior to surgical exploration, revealing an enlarged epididymis suspicious for malignancy^4^^,^^5^ (Table 1).

**Table 1:** Summary of case reports on epididymal cavernous haemangioma.

	Year	Country	Age	Duration of symptoms	Treatment
Epididymal cavernous haemangioma					

Karry^4^	2018	Tunisia	56 years	3 years	Orchidectomy after epididymectomy was attempted
Chetty^5^	1993	Australia	44 years	4 weeks	Orchidectomy
			50 years	Sudden	Epididymectomy
ˆppTesticular + epididymal cavernous haemangioma					

Robertson^3^	1954	U.S.A	15 years	5 days	Orchidectomy
Rosenthal^6^	1946	U.S.A	3 months	3 months	Orchidectomy

Although scrotal exploration was prompt in our case, the secondary intratesticular haemorrhage significantly impaired the ongoing viability of the testis and consequently could not be spared. Could epididymectomy have been performed if the patient had presented earlier? A review of the literature identified one case in which epididymectomy was successfully performed.^5^ That case involved a 50-year-old patient who presented with acute right testicular pain and underwent epididymectomy two weeks after symptom onset, following two weeks of antibiotics for a presumed infected epididymal cyst.^5^ The difficulty of performing epididymectomy was described by Karry et al.,^4^ who reported a 56-year-old patient who presented three years after symptom onset. An attempt at epididymectomy was unsuccessful due to intense inflammation.^4^

In our case, histopathology revealed normal spermatogenesis, likely due to the prompt surgical intervention. In contrast, Chetty et al. reported a 44-year-old patient who presented four weeks after noticing left testicular swelling.^5^ Microscopic examination in that case revealed hypospermatogenesis, likely due to raised testicular temperature secondary to the haemangioma.

Another paediatric case of epididymal and testicular haemangioma was described by Rosenthal et al..^6^ This involved a 3-month-old infant with recurrent hydrocoele. Scrotal exploration was performed due to concern for trauma to the testis from repetitive aspiration of the hydrocele. Intraoperatively, the testis appeared bluish black with an indurated and thickened epididymis. Microscopic examination revealed extensive haemorrhage throughout the testicle, extending to the epididymis. A diagnosis of cavernous haemangioma involving the testis and epididymis was made.

The International Society for the Study of Vascular Anomalies (ISSVA) classifies vascular anomalies into three main categories: vascular tumours, vascular malformations, and potentially unique vascular anomalies.^7^ According to the ISSVA 2025 classification, cavernous haemangiomas are categorised as a vascular malformation. Unlike vascular tumours, which are characterized by endothelial hyperplasia, vascular malformations feature flat endothelial linings.^8^

Vascular malformations are congenital and arise due to developmental errors during embryogenesis. Their growth typically matches the rate of the individual's overall growth.^8^ A retrospective study involving 115 patients with vascular malformations reported a mean age of diagnosis of 6.7 years.^9^ Several syndromes are linked to vascular malformations, including Klippel-Trenaunay syndrome, Maffucci syndrome, and Proteus syndrome.^10^

Cavernous haemangiomas most frequently occur in the brain 2. A number of factors have been associated with an increased risk of bleeding in cases of cerebral cavernous haemangioma, such as a family history of the condition,^11^ mutations in the **CCM3** gene,^12^ the presence of developmental venous anomalies (DVA),^13^ and symptomatic lesions.^14^ Genital haemangiomas are rare. A literature review of testicular haemangiomas identified only 55 reported cases to date.^15^ Histologically, testicular haemangiomas are classified into four categories: cavernous haemangioma, capillary haemangioma, histiocytoid haemangioma, and papillary endothelial hyperplasia.^16^

In conclusion, we report a case of epididymal cavernous haemangioma in a teenager, with a presentation resembling testicular torsion. Prompt scrotal exploration was performed in our case. Although histological examination later revealed normal spermatogenesis, the testis was not viable due to intratesticular haemorrhage, justifying orchidectomy. A review of the literature shows that the onset of symptoms can be acute or insidious, mimicking a variety of testicular conditions, including torsion, infection and cancer. As a rare condition, our case serves an educational purpose for clinicians encountering similar presentations.

## CRediT authorship contribution statement

**Siyu Huang:** Writing – original draft. **Thomas McLean:** Writing – review & editing, Data curation, Resources. **Annjaleen Hansa:** Data curation, Writing – review & editing. **Jordan Santucci:** Writing – review & editing. **Andrew Silagy:** Writing – review & editing. **Lydia Johns-Putra:** Writing – review & editing.

## Consent to publish

Informed consent to publish the case has been obtained from both patient and parents.

## Declarations

The authors declare that they have no competing interests.

## Funding sources

This research did not receive any specific grant from funding agencies in the public, commercial, or not-for-profit sectors.

## Declaration of competing interest

The authors declare that they have no known competing financial interests or personal relationships that could have appeared to influence the work reported in this paper.

## List of abbreviations

DVA, developmental venous anomalies.

## References

[1] Priemer D.S., Trevino K., Chen S., Ulbright T.M., Idrees M.T. (2017 Sep). Paratesticular soft-tissue masses in orchiectomy specimens: a 17-year survey of primary and incidental cases from one institution. Int J Surg Pathol.

[2] Cavernous Malformation [Internet] National organization for rare disorders. https://rarediseases.org/rare-diseases/cavernous-malformation/.

[3] Robertson J.W., Palitz S. (1954 Nov). Hemangioma of testis and epididymis. J Urol.

[4] Karray O., Ben Chehida M.A., Sellami A. (2018 Nov 6). A rare scrotal tumor: epididymal cavernous hemangioma. Case Rep Urol.

[5] Chetty R. (1993 Apr). Epididymal cavernous haemangiomas. Histopathology.

[6] Rosenthal A.A. (1946 May). Hemangioma of the testis in an infant. J Urol.

[7] International society for the study of vascular anomalies. https://www.issva.org/classification.

[8] Cohen M.M. (2006 Oct 1). Vascular update: morphogenesis, tumors, malformations, and molecular dimensions. Am J Med Genet.

[9] Vogel S.A., Hess C.P., Dowd C.F. (2013 Sep). Early versus later presentations of venous malformations: where and why?. Pediatr Dermatol.

[10] Cohen M.M. (2002 Apr 1). Vasculogenesis, angiogenesis, hemangiomas, and vascular malformations. Am J Med Genet.

[11] Josephson C.B., Salman R.A.-S., Rigamonti D. (2011). Cavernous Malformations of the Nervous System.

[12] Shenkar R., Shi C., Rebeiz T. (2015 Mar). Exceptional aggressiveness of cerebral cavernous malformation disease associated with PDCD10 mutations. Genet Med.

[13] Rigamonti D., Spetzler R.F. (1988). The association of venous and cavernous malformations. Report of four cases and discussion of the pathophysiological, diagnostic, and therapeutic implications. Acta Neurochir.

[14] Mathiesen T., Edner G., Kihlström L. (2003 Jul). Deep and brainstem cavernomas: a consecutive 8-year series. J Neurosurg.

[15] Marino F., Lorusso G., Gandi C., Ragonese M., Pierconti F., Sacco E. (2025 Feb). Epithelioid (Histiocytoid) hemangioma of the testis: a case report and literature review of a rare benign tumor. Urologia.

[16] Mazal P.R., Kratzik C., Kain R., Susani M. (2000 Aug). Capillary haemangioma of the testis. J Clin Pathol.

